# Vacuum and Electromagnetic Fields Treatment to Regenerate a Diffuse Mature Facial Scar Caused by Sulfuric Acid Assault

**DOI:** 10.3390/bioengineering9120799

**Published:** 2022-12-13

**Authors:** Sheila Veronese, Bruno Brunetti, Anna Maria Minichino, Andrea Sbarbati

**Affiliations:** 1 Department of Neuroscience, Biomedicine and Movement Sciences, University of Verona, 37134 Verona, Italy; 2 Dermatological Clinic Brunetti, 84129 Salerno, Italy

**Keywords:** acid attack, burn, scar, V-EMF, regenerative treatment

## Abstract

Acid attacks are on the rise, and they cause extensive and deep burns, especially on the face. The treatments used to improve the aesthetic, functional and social impact of non-acid scars do not always prove useful for acid scars. This article reports the case of a woman with an extended, mature, acid facial scar, caused by sulfuric acid assault, treated with a recent new procedure that combines the application of vacuum and electromagnetic fields. Before and after the treatment, the aesthetic appearance, and motor function of the face and neck were evaluated, as well as the level of hydration, the amount of sebum, the elasticity, and the pH of the skin. The improvements highlighted after the treatment of the aesthetic and functional characteristics of the face and neck, and of the physical parameters of the skin seemed to indicate that this particular treatment induces tissue regeneration, even in the nerve component. However, it is evident that the rehabilitation pathways of facial wounds and scars must be personalized, and must include continuous psychological support for the patient.

## 1. Introduction

The data on the increase in the number of chemical attacks are alarming [1,2,3,4,5]. Mortality associated with these actions is very high [4,5,6,7,8], and, considering that the victim is often attacked in the face, the sequelae completely destroy her/his life because the damage is aesthetic, functional and social [9,10,11,12,13,14,15,16,17].

Among the acids most used to perform the attacks is sulfuric acid, which on the skin has a double action, both chemical and thermal, due to its properties [4,7,18,19]. The effects of prolonged and massive exposure to this acid, if the victim survives, are both disfiguring and dysfunctional [4,5,7,8,20,21,22] due to the long healing times of the wounds and the fact that numerous reconstructive surgeries are often necessary [7,23,24].

There are numerous more or less invasive treatments aimed at improving residual aesthetic stigmatizations and facial dysfunctions (Table 1, [25,26,27,28,29,30,31,32,33,34,35,36,37,38,39,40,41,42,43]), but an optimal treatment has not yet been identified [44,45]. In fact, although many interventions appear promising, sometimes the results reported in the literature are conflicting [32,33,34,35], or interventions suitable for some types of scars are not effective for others [29,43,44,45]. This may depend on the extreme variability of individual situations, for each of which a personalized medical-surgical-rehabilitation path should be identified.

In this report we present the case of a woman with a mature scar, extending to the whole face, caused by a sulfuric acid attack and treated with a new recent multi-technique procedure. The latter is a form of therapy, generally used for small scars [46], which combines the application of vacuum and electromagnetic fields (V-EMF). 

### V-EMF Treatment

In V-EMF therapy, a medium frequency electromagnetic field is applied in a vacuum regime, directly to the affected area. The waves used are radio waves with a frequency range of 450–2000 kHz (the frequency range generally used in resistive-capacitive diathermy is 450–1200 kHz). Energy is transferred to the tissues in a capacitive way, by means of a single metal electrode, suitably shielded by insulating material. The second conductive plate of the capacitor is given by the body tissue, and this implies that the electromagnetic charge is concentrated near the isolated electrode, i.e., in the superficial tissues [47,48,49].

From the biomedical point of view, an endogenous diathermic effect and a magneto-mechanical effect are simultaneously induced on the treated tissues. The thermal effect is due to the transformation of the kinetic energy of the ions, which move due to electromagnetic waves, into heat (Joule effect) [50,51,52]. The magneto-mechanical effect is linked to the piezoelectricity of some tissues, i.e., to their ability to mechanically alter their structure following a magnetic stress [53,54].

The first effect determines an increase in metabolic reactions. There is an increase in microcirculation, with a consequent increase in the number of gaseous exchanges between blood and tissues. The catabolic products are drained more quickly and the diapedesis of granulocytes, macrophages, and of the cells involved in inflammatory and reparative processes increases. In addition, the “cell killing” effect of senescent and damaged cells occurs [48,49,55,56,57,58]. The rise in temperature extends deeper [48,49,55], although the actual amount progressively decreases as it deepens from the surface of the skin [47,58]. However, this involves an overall analgesic effect [47,49,56,59], and consequently a well-being after therapy, with muscle relaxation, an increase in muscle flexibility [60,61], a reduction in pain associated with movements, and an increase in the elasticity of the connective tissue [49,56,61,62].

The second effect occurs mainly at the level of connective tissue, which is the body tissue with the most significant piezoelectric characteristics. The structural deformation of this tissue favours the resolution of fibrotic states, and the rebalancing of the extracellular matrix [63,64].

The combination of the two effects improves the repair of all involved tissues, and wound healing. We can speak of a real regenerative effect, given that there is an overall tissue regeneration [49,58,59], including that of the neural component [65,66,67,68].

The application of electromagnetic waves in a vacuum regime (100–150 millibar) amplifies the effects that these waves induce on the tissues. In particular, the vacuum appears to play a fundamental role in the restructuring of the extracellular matrix, since the induced mechanical stimulus activates the endothelial cells, the fibroblasts and the cutaneous myofibroblasts [69,70].

## 2. Materials and Methods

### 2.1. Case

We present the case of a 63-year-old female, attacked with sulfuric acid on 28 May 2012 and hospitalized from 28 May 2012 to 2017 at the Cardarelli Hospital in Naples, Italy. Here she underwent 27 surgeries for the reconstruction of the face and neck, through autologous skin grafts taken from different parts of the body, and 3 autologous lipofilling procedures. After the surgeries, she did not use an elastic compression mask at home. From the event, the patient has been followed from a psychological point of view by the Association “Women for Women Against Violence”.

After 10 years from the attack, on the face and neck, she visually showed retraction of the skin and marked dyschromia. Evident were the flattening of the nose, presumably due to the retraction of the peri-labial tissue, and the deformation of the neck, with the absence of the typical right-angled conformation of the platysma and the presence of a diagonal line shape.

Face and neck showed hardening and thickening of the skin surface, and loss of sensation, with absence of both slight tactile perception and pinching.

From a functional point of view, the deformation of the nose involved the reduction of the nasal rostrums, resulting in the need to breathe through the mouth. The deformation of the neck reduced its motility. The rotational movement of the head appeared limited and sometimes painful on the side opposite to that of rotation. The retraction of the skin tissue at the level of the face induced traction of the lower lip with consequent involuntary opening of the mouth, in case of extension of the head.

### 2.2. Methods

The patient underwent V-EMF treatment, that was delivered according to the Biodermogenesi^®^ method, using the Bi-one^®^ LifeTouchTherapy device (Expo Italia Srl, Florence, Italy), and with the protocol already detailed in Veronese et al. [46]. Specifically, the subject underwent a cycle of 12 sessions, lasting 25 min each, with the frequency of one session a week, in the period April–May 2022. The vacuum was applied at 100–150 millibars. The frequency used for the generation of the EMF varied between 0.5 and 2 MHz, i.e., the supplied power was on average 4 W. The variability was linked to an automatic self-regulation system of the device, linked to an automatic feedback control of the quantity of energy absorbed by the skin. This amount is, in turn, related to the thickness of the treated skin.

A neutral alcohol-based cleanser was used on the skin before starting the procedure. Before each session, the operator identified the main fibrous excerpts present by palpation. In an initial phase lasting about 15 min, the handpiece was applied to the fibrotic excerpts, first parallel to their progression (10 min), to obtain a progressive softening action, and subsequently (5 min), tangentially. In the remaining 10 min, the handpiece was passed over the whole face and neck, to generalize the action.

Before the first treatment session (T0) and a week after the last session (T1) the level of hydration, the quantity of sebum, the elasticity, and the pH of the skin were measured at the level of the center of the forehead, of the left and right cheekbones, and in the center of the chin. To make the measurements the Skin Plus^®^ device (Lemi s.r.l., Casalbuttano and Uniti, Italy) was used. Measurement was performed 2 times per test type and per location. The value obtained from the first survey was considered valid if confirmed by a second survey, with a tolerance of 5%. In the presence of greater differences, the average of the 2 measured values was taken.

At T0 and T1, photographs of the forehead, middle third of the face, nose in profile, and lower third of the face were taken for a qualitative evaluation of the effects of the treatment. A Nikon D500 camera was used, at a distance of 1 m from the patient, with artificial light.

## 3. Results

The level of hydration, the amount of sebum, the elasticity, and the pH of the skin recorded at T0 and T1 are shown in Table 2. For all tests and all sites, the 2 measurements taken differed by 2–3%. Therefore, the first value detected was considered valid.

After the treatment, 2 of the 4 tested parameters did not normalize in the forehead area. The level of skin hydration remained practically unchanged, while the amount of sebum produced exceeded the upper limit of the norm. The latter data was common to all the facial areas considered.

In the cheek there was an increase above the normal range of skin elasticity. Therefore, even for the cheek 2 out of 4 parameters did not normalize.

**Figure 1:**
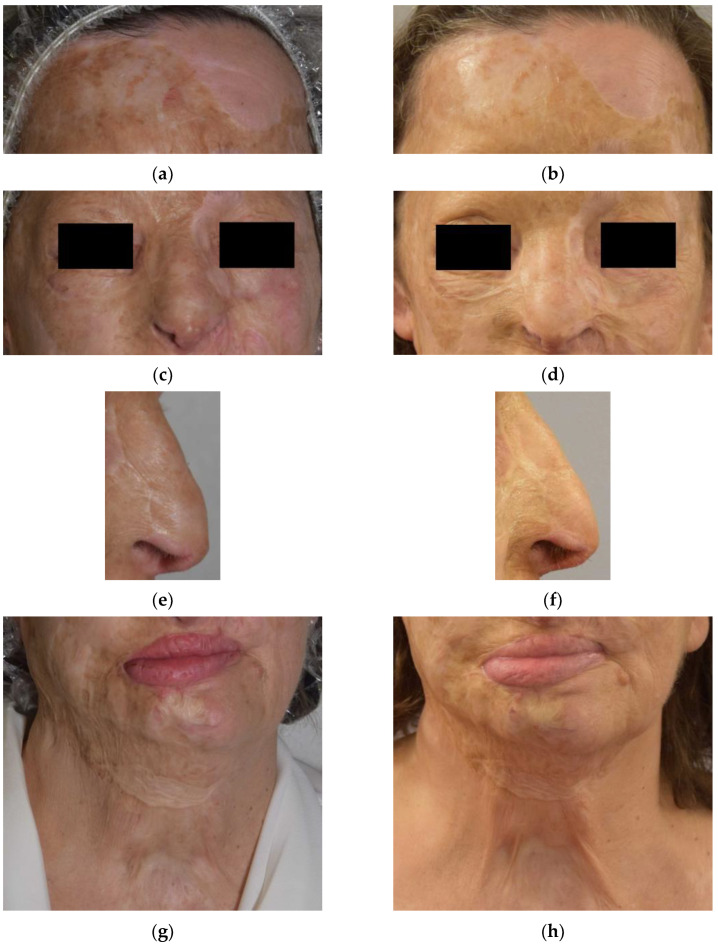
Photographs just before, and 1 week after the V-EMF treatment of the forehead (**a**,**b**), middle third of the face (**c**,**d**), nose (**e**,**f**), and lower third of the face (**g**,**h**).

On the surface, the skin appeared less tight. Some scar furrows appeared smooth. On palpation, the fibrotic tissue mainly present in the nose, chin and neck was softer and less adherent. The structure of the nose and the lower middle third of the face were particularly reshaped, presenting a more natural conformation. The photographic comparison at T0 and T1 is shown in Figure 1.

Finally, it should be noted that during the single treatment sessions the subject did not report any discomfort. No side effects or pain were reported during treatment and at T1.

## 4. Discussion

In cases of acid assault, the face is generally the most affected part of the body [3,4,5]. The first necessity when a patient arrives at the hospital with this type of injury is always to save her/his life. Only when the survival of the subject has been guaranteed is it possible to intervene to try to preserve all the functions of the face (primarily the visual one), promote rapid healing, and minimize visual stigmatization [19,71]. 

The precocity of treatments seems to indicate a better functional and aesthetic wound healing, with the formation of less extensive and shallow scars [24,72,73]. Unfortunately, timeliness is not always possible, as in the case described in this study, where healing times were very long. In addition, there are people who have old scars and/or who have not benefited from the various treatments performed [44,45]. In these cases, a long time passes after the initial injury. Thus, a treatment may, instead of promoting regeneration (as in the proliferative/modulating phase of healing), cause a resumption of an inflammatory process. This process may ultimately culminate in a major proliferative phase, but first by inducing a major distress phase in the treated subject.

The V-EMF procedure has the advantage of being completely non-invasive and the results obtained with the treatment of small scars are highly encouraging [46]. The application of this treatment on an extensive scar like that of the case described, although not a bet, did not guarantee the results obtained. The latter are extremely interesting, especially from a functional point of view. It should be noted that even the aesthetic results achieved are not trivial. For instance, the regeneration of the nose is evident, both in the skin and in the redefinition of the nasal rostrums (Figure 1c–f). 

The reappearance of tactile sensitivity and the improvement of cranio-cervical motility further highlight the regeneration of the tissues damaged by the burn, a regeneration that also includes the neural components. Furthermore, the changes in skin characteristics after treatment (Table 2) demonstrate the regeneration of the skin texture and the normalization of many of its properties. The authors believe that the fact that sebum production increases beyond normal levels underscores the skin’s strong natural response to treatment. And, therefore, it is not seen as a negative datum. The greater elasticity of the cheek could be linked to the greater amount of adipose tissue generally present in this area.

Given the results obtained, in the absence of any discomfort, it can be concluded that the procedure did not reactivate a situation of pain and inflammation, but a tissue regenerative activation was directly induced. This is particularly interesting also considering the age of the treated subject, an age in which the regenerative capacity in general, and of the face in particular, can be the subject of discussion.

In the literature there are not many analyses relating to this technique for the treatment of scars, given that it was recently introduced. In the two works that describe it [46,74], for ethical reasons, no invasive analyzes (e.g., biopsy analysis) were performed to evaluate the effectiveness of the method. The outcomes of the various treatments were evaluated by non-invasive measurements and were considered satisfactory in both studies. Nevertheless, in three studies V-EMF therapy was applied to stretch marks, often referred to as atrophic scars [75,76,77]. Two of these studies also reported histological evaluations of biopsies taken after the treatments [76,77]. These evaluations highlighted a tissue reorganization with restoration of the original volume. A neoformation of collagen shoots and elastin fibers was observed, with restructuring of the basement membrane, and of the extracellular matrix underlying the striae. Although the degree of tissue degeneration linked to chemical burn scars is undoubtedly not comparable to the structural alteration linked to the presence of a stretch mark, the reported observations highlight a regenerative reaction in the tissue treated with V-EMF therapy. This is certainly an important fact.

Studies in the literature described different types of treatment for burn cases, with excellent results for some aspects and absent or negative results for others [31,35,37,40,41]. One wonders if the combination of several different treatments can be significant for the full aesthetic and functional recovery, and for the improvement of all aspects of the quality of life of burned subjects. For this particular category of patients, it seems not only useful, but necessary to define a personalized therapeutic path. V-EMF treatment, which is a multi-technique procedure, appears to respect this principle. Perhaps in these particular subjects, with extensive and deep burn scars and the presence of painful fibrotic shoots, it may be necessary for patients to undergo multiple cycles of V-EMF therapy, to progressively resolve/improve their pathological state.

Finally, it is essential not to forget the fact that people with scars due to acid attacks are first of all victims, which must be followed from a psychological point of view, even during treatments, given the strong emotional impact that aesthetic-functional recovery can have [78,79,80]. 

## 5. Conclusions

Undoubtedly, the application of V-EMF treatment to a greater number of subjects is necessary to confirm the results obtained for the subject of this report. Considering this case, it can be concluded that V-EMF therapy appears to be beneficial for mature and widespread scars in burn outcomes. It is very promising that both aesthetic and functional recovery have been observed. 

## Figures and Tables

**Table 1:** Most common scar treatments.

Invasiveness	Type	References
Non-invasive	Silicone sheet coating	[25]
	Topical treatment	[26,27]
	Application of mesenchymal stem cells in tissue scaffolds	[28]
Minimally invasive	Corticosteroid injection	[29]
(principally injectable therapies)	Botox injection	[30]
	Mesenchymal stem cells injection (principally obtained from fat grafting)	[31,32,33]
	Hyaluronic acid filler	[34,35]
Invasive	Cryotherapy (generally applied after surgical excision)	[36]
Others (more/less invasive)	Laser - minimally invasive—non-ablative - invasive—ablative	[37,38,39]
	Shock wave therapy	[40]
	Radiofrequencies application	[41]
	Microneedling	[42]
	Dermabrasion (combined with regenerative agents)	[43]

**Table 2:** Skin parameters.

	Forehead		Right Cheekbone		Left Cheekbone		Chin	
	T0	T1	T0	T1	T0	T1	T0	T1
Hydration Level	12	18 *	32	46	35	86	34	73
normal value	>44/100							
Sebum Quantity	27	43 **	25	65 **	27	70 **	29	63 **
normal value	<40/100							
Skin Elasticity	18	48	16	45	16	44	15	63 **
normal value	>20/50							
Skin pH	3.7	4.9	3.6	5.0	3.6	4.8	3.2	4.9
normal range	4.1–5.8							

* increase below normal levels; ** increase above normal levels.

## Data Availability

All the data used for this study are present in the text.
